# Terpene-Enriched Nitazoxanide-Loaded Chondrosomes: Aerodynamic Characterization and In Silico Evaluation of Antiviral Activity

**DOI:** 10.3390/ph19050702

**Published:** 2026-04-29

**Authors:** Rofida Albash, Anroop B. Nair, Mohamed A. Morsy, Katharigatta N. Venugopala, Pottathil Shinu, Amira B. Kassem, Asmaa Saleh, Moaz A. Eltabeeb

**Affiliations:** 1 Department of Pharmaceutics, College of Pharmaceutical Sciences and Drug Manufacturing, Misr University for Science and Technology (MUST), Giza 12585, Egypt; 2 Department of Pharmaceutical Sciences, College of Clinical Pharmacy, King Faisal University, Al-Ahsa 31982, Saudi Arabia; momorsy@kfu.edu.sa (M.A.M.); kvenugopala@kfu.edu.sa (K.N.V.); 3 Department of Biomedical Sciences, College of Clinical Pharmacy, King Faisal University, Al-Ahsa 31982, Saudi Arabia; spottathail@kfu.edu.sa; 4 Department of Clinical Pharmacy and Pharmacy Practice, Faculty of Pharmacy, Damnhour University, Damanhur 22516, Egypt; 5 Department of Pharmaceutical Sciences, College of Pharmacy, Princess Nourah Bint Abdulrahman University, P.O. Box 84428, Riyadh 11671, Saudi Arabia; 6 Department of Industrial Pharmacy, College of Pharmaceutical Sciences and Drug Manufacturing, Misr University for Science and Technology (MUST), Giza 12585, Egypt

**Keywords:** aerodynamic simulation, drug repurposing, nitazoxanide, chondroitin sulfate, in silico study

## Abstract

**Background/Objectives**: This investigation aims to assess the potential for repurposing nitazoxanide (NIT) as a treatment for COVID-19. NIT was loaded into terpene-enriched chondrosomes (TECs) to assess its anti-hCoV-19 activity through pulmonary delivery. **Methods:** NIT-TECs were then fabricated utilizing the ethanol injection method. Using a D-optimal design, the effects of factors on entrapment efficiency (EE%), particle size (PS), and zeta potential (ZP) were determined, and the optimal formulation was selected. **Results:** The optimum TEC exhibited an EE% of 98.87 ± 0.69, a PS of 129.43 ± 5.43 nm, a polydispersity index (PDI) of 0.433 ± 0.022, and a ZP of −25.99 ± 0.99 mV. The optimum TEC was lyophilized to attain a dry powder. Further, the differential scanning calorimetry test confirmed that NIT was transformed from crystalline to amorphous form inside the optimum TEC. In addition, the mucoadhesion test confirmed the ability of the optimum TECs to adhere to pulmonary tissues. Additionally, NIT binding to the active site of SARS-CoV-2 enzymes was investigated using in silico analysis. When compared to NIT, the aerodynamic characteristics of the lyophilized optimum TECs employing the cascade impactor showed superior residence in the lungs. **Conclusions:** These findings suggest that loading NIT into TECs enhanced its antiviral activity, as indicated by the in vitro cytotoxicity study. Overall, the results point to NIT-loaded TECs as a potentially effective pulmonary delivery system for COVID-19 treatment.

## 1. Introduction

Coronavirus disease (COVID-19) continues to pose a universal health hazard due to its ability to induce severe clinical manifestations [1]. Various surprising old medications are being repurposed in the COVID-19 era to identify new alternatives that have affordable and effective qualities against SARS-CoV-2. The antiparasitic medication nitazoxanide (NIT) is one of these chosen medications. NIT has been reported for the treatment of influenza and is frequently used to treat Giardia intestinalis and Cryptosporidium parvum [2]. Additionally, NIT has some effectiveness against the hepatitis C virus and is beneficial in managing the hepatitis B virus [3]. Moreover, by activating interferon, restoring innate immunity, preventing the production of pro-inflammatory cytokines, suppressing the mammalian target of rapamycin, and inducing autophagic cell death, NTZ can lessen SARS-CoV-2-induced inflammatory reactions. Furthermore, it can prevent the production of oxidative stress, which is linked to acute lung injury, acute respiratory distress syndrome, and multi-organ damage and results in a cytokine storm [4].

Delivery systems that use nanotechnology have evolved as promising platforms for enhancing antiviral therapies [5], offering targeted delivery, improved cellular uptake, and the potential to overcome biological barriers [6]. Natural bioactive compounds, particularly terpenes, have additionally shown antiviral and anti-inflammatory features, making them attractive functional excipients for pulmonary formulations for infection [7].

Several studies supported the anti-inflammatory, antimicrobial [8], and antiviral [9], properties of 1,8-cineole. Also, a clinical trial employing the monoterpene 1,8-cineol demonstrated mucolytic and bronchodilation activities [10]. Further, previous investigations stated that terpenes such as fenchone possess antiviral bioactivities [7].

Likewise, sulfated polysaccharides such as chondroitin sulfate (CS) exhibit noteworthy antiviral effects through interference with viral attachment and entry [11], including reported activity against betacoronaviruses [12]. Further, it has been reported that sulfated polysaccharides inhibit infection with human viral pathogens such as papilloma [13] and influenza A [14] viruses. By preventing viral attachment through steric and electrostatic interference with viral surface proteins, CS reduces cell entrance and limits viral infection. By encouraging the recruitment of immune cells and controlling inflammation, its interaction with the CD44 receptor further strengthens antiviral defense. Therefore, the CS–CD44 axis combines immunomodulatory effects with direct viral suppression [15]. Incorporating such bioactive components within nanocarriers may provide synergistic enhancement of antiviral activity.

It is worth mentioning that ceramide-containing vesicular systems have recently gained attention for pulmonary drug delivery, with evidence suggesting that ceramides may hinder viral attachment and replication [16]. A recent publication by Albash et al. utilized ceramide-enriched vesicles as a carrier for pulmonary delivery of moxifloxacin hydrochloride against COVID-19, and the authors highlighted the importance of utilizing ceramide presence and proved its efficacy in silico against COVID-19 [17].

Collectively, terpenes, CS, and ceramides represent promising multifunctional components that may augment the antiviral performance of NIT when co-formulated into a nanoscale pulmonary delivery system. Based on this rationale, the present study aims to develop and optimize terpene-enriched chondrosomes (TECs) loaded with NIT as a novel inhalable nanocarrier for COVID-19 therapy. A D-optimal design was employed to investigate the impact of formulation variables and to identify the optimal TEC formulation. The optimized system was subsequently characterized by aerosolization behavior, mucoadhesion, and in vitro antiviral efficacy against hCoV-19-infected Vero E6 cells. Furthermore, computational studies were performed to explore the interaction of NIT with key SARS-CoV-2 active sites. This combined approach aims to boost the pulmonary delivery and antiviral activity of NIT, offering a promising nanotechnological strategy for the management of COVID-19.

## 2. Results

### 2.1. Optimization of TECs by Applying D-Optimal Design

The experimental design utilizing response surface methodology (RSM) focuses on identifying optimal variables for a certain response aim while minimizing the number of experiments conducted. Furthermore, RSM has been employed to assess the correlation among several independent variables and ascertain the optimal circumstances necessary to get a certain desired response [18]. A D-optimal design was employed using Design-expert^®^, (Version 13) producing 13 runs. A linear model was found to best fit all three responses, showing adequate precision values of 24.74, 32.73, and 76.87 for entrapment efficiency (EE%), particle size (PS), and zeta potential (ZP), respectively, confirming acceptable signal-to-noise ratios (Table 1). The linear model was selected by Design-expert^®^ software as the best fit based on statistical criteria, including model significance and goodness-of-fit parameters.

### 2.2. EE% and Drug Loading (DL%) Capacity

The amount of NIT in the EE% calculation was quantified using a spectrophotometer. The method demonstrated good linearity (y = 0.1012x − 0.0467) in the range of 2.5–20 µg/mL, with a high correlation coefficient (r^2^ = 0.999). The specificity of the method was confirmed by the absence of interference from formulation excipients at the selected wavelength.

The selected factorial model was highly significant (*p* < 0.0001), as supported by ANOVA. The model’s F-value of 72.91, low *p*-value (*p* < 0.0001), and R^2^ (0.986) and adjusted R^2^ (0.972) values indicate that 98.65% of the variability in %EE was explained by the model, demonstrating excellent fit. Additionally, the predicted R^2^ (0.882) was in close agreement with the adjusted R^2^ (difference < 0.2), reinforcing model validity. The adequate precision value (24.7470) exceeded the threshold of four, confirming a strong signal-to-noise ratio and reliable model navigation within the design space.

**Figure 1:**
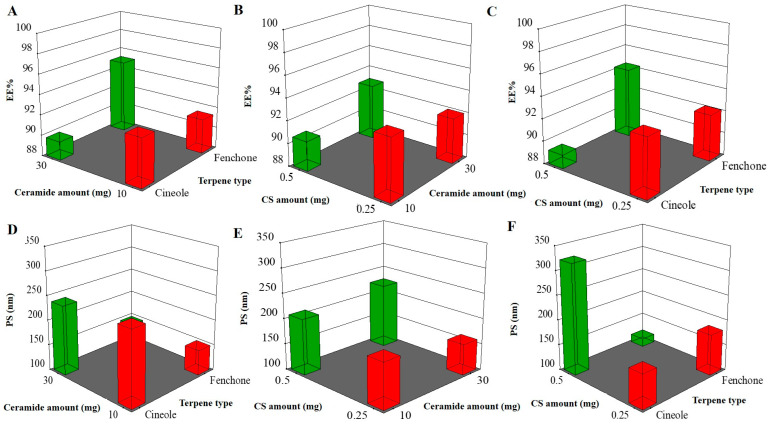
Effect of formulation variables on EE% (**A**–**C**) and PS (**D**–**F**). EE%, entrapment efficiency; PS, particle size; and CS, chondroitin sulfate.

One of the main factors used to assess the prepared vesicle’s performance is its ability to hold an acceptable amount of medication (Figure 1). The EE% values ranged from 89.24 ± 0.74 to 98.87 ± 0.69% and DL% ranged from 57.57 ± 3.98 to 63.78 ± 1.45% (Table 2). For terpene type (X_1_), the factor shows significance with a *p*-value of 0.0004. It was found that EE% increased when using cineole instead of fenchone. Given that NIT is a hydrophobic medication, it is anticipated that the EE% will rise with an increase in the lipophilic nature of the terpene contained in the vesicles. In the present investigation, cineole exhibited more hydrophobicity than fenchone, which contains a ketone group, making it more hydrophilic, with log *p*-values of 2.82 for cineole and 2.13 for fenchone [19]. The previous findings agreed with those of El-Nabarawi et al. during their preparation of dapsone-loaded invasomes [20]. For ceramide amount (mg) (X_2_), the factor was not significantly related to the response, with a *p*-value of 0.1189. Regarding CS concentration (X_3_), by increasing the CS amount, the EE% decreased. The previous literature stated that CS is hydrophilic in nature [21]; hence, increasing the number of hydrophilic counterparts might aid in increasing the hydrophilicity of the developed system and enhance NIT leakage from the nanocarriers. The interaction terms’ coefficient values (AB, AC, and BC) show that the formulation variables’ combined effects have a significant effect on EE%. Although there appears to be some correlation between the predictors, the variance inflation factor (VIF) values (>1) stay within acceptable bounds, suggesting no significant multicollinearity.

### 2.3. PS

The reduction of the PS to a nanometric range has been suggested to further enhance the potential of carriers for lung mucosal delivery of various bioactives [22]. The selected factorial model was highly significant (*p* < 0.0001), as supported by ANOVA. The model’s F-value of 123.64, low *p*-value (*p* < 0.0001), and R^2^ (0.992) and adjusted R^2^ (0.984) values indicate that 99.2% of the variability in PS was explained by the model, demonstrating excellent fit. Additionally, the predicted R^2^ (0.885) was in close agreement with the adjusted R^2^ (difference < 0.2), reinforcing model validity. The adequate precision value (32.7376) exceeded the threshold of 4, confirming a strong signal-to-noise ratio and reliable model navigation within the design space. As shown in Figure 1, the PS of TECs ranged from 108.82 ± 0.82 to 340.49 ± 3.49 nm (Table 2). For the terpene type (X_1_), *p* < 0.0001, it was shown that cineole produced a larger PS than fenchone. The molecular weight (MWT) discrepancies between the two oils may have contributed to the earlier findings, as cineole had a higher MWT than fenchone. Other research confirmed our findings and showed that bigger MWT excipients may result in larger PS nanocarriers [23]. For ceramide amount (mg) (X_2_), the factor shows no significance, with a *p*-value of 0.314. For CS concentration (X_3_) (*p* = 0.002), ANOVA results showed that when increasing the concentration of CS, the PS markedly increases. The previous literature stated that CS, being a hydrophilic polymer with a long chain, as previously stated, tends to form larger PS nanovesicles [24], as the polymer chains can act as a framework around which nanoparticles form and might absorb water on their surface, promoting a larger PS [25]. The interaction terms’ coefficient values (AB, AC, and BC) show a favorable combined effect on particle size (PS), indicating that the formulation variables interact rather than work separately. Although there is a relatively small correlation across the predictors, the variance inflation factor (VIF) values (>1) stay within acceptable limits, suggesting no major multicollinearity. In contrast to previous interaction factors, the combined effect of terpene type and CS concentration demonstrated a noteworthy inverse relationship with PS.

### 2.4. Polydispersity Index (PDI)

The PDI was measured to evaluate dispersion homogeneity. Values approaching zero signify a monodisperse, highly uniform system, whereas high values approaching one indicate highly polydisperse dispersions [26]. In our study, the PDI values of the TECs ranged from 0.226 ± 0.009 to 0.433 ± 0.022, indicating a degree of heterogeneity in PS, yet still inside the valid range for its estimation through dynamic light scattering (Table 2) [27]. Statistical analysis of the data via ANOVA showed that none of the investigated factors had a statistically significant impact on PDI values [28,29].

### 2.5. ZP

**Figure 2:**
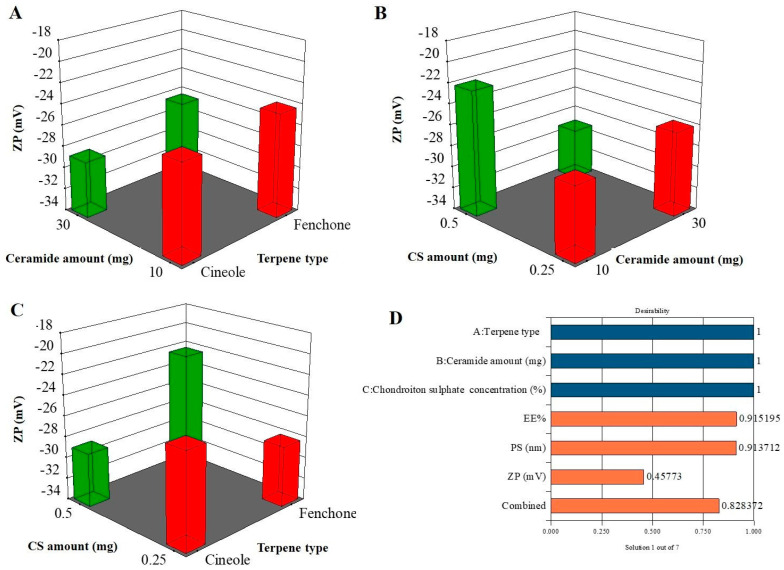
Effect of formulation variables on ZP (**A**–**C**) and desirability for the optimum formulation (**D**). Blue bars: Independent formulation variables; Orange bars: Dependent response variables. EE%, entrapment efficiency; PS, particle size; ZP, zeta potential; and CS, chondroitin sulfate.

ZP, which calculates the charges obtained by vesicles, is a measure of a system’s stability. Particles with a ZP less than −15 mV or more than 15 mV are expected to be stable [30]. The selected factorial model was highly significant (*p* < 0.0001), as supported by ANOVA. The model’s F-value of 527.59, low *p*-value (*p* < 0.0001), and R^2^ (0.998) and adjusted R^2^ (0.996) values indicate that 99.8% of the variability in ZP was explained by the model, demonstrating excellent fit. Additionally, the predicted R^2^ (0.99) was in close agreement with the adjusted R^2^ (difference < 0.2), reinforcing model validity. The adequate precision value (76.8745) far exceeded the threshold of four, confirming a strong signal-to-noise ratio and reliable model navigation within the design space. The ZP values (Figure 2A–C) of the formulated TECs ranged from −20.09 ± 0.89 to −33.19 ± 0.37 (Table 2). The negative charge of particles might be related to hydroxyl, carboxyl, and sulfonic acid groups, which implies that negatively charged CS is allocated on the surface [31]. For terpene type (X_1_) (*p* < 0.0001), cineole showed a larger ZP than vesicles prepared with fenchone. As previously stated in the PS section, cineole showed a larger PS than fenchone-containing vesicles, and the authors attributed the difference to MWT. It is worth mentioning that a previous investigation supported that the MWT increases the ZP of the vesicles [32].

For ceramide amount (mg) (X_2_) (*p* < 0.0001), the factor was significant; it was found that by increasing the ceramide amount, the negative value of ZP increased. It is worth noting that augmenting the ceramide content up to 30 mg resulted in a more viscous formulation [33]. Previous studies stated that as the viscosity of the colloidal system increased, the ZP values increased to more negative values, which was in accordance with our outcomes [34]. For CS concentration (X_3_) (*p* = 0.08), ANOVA results showed that the factor shows no significance. ZP is significantly impacted by the combined effects of formulation variables according to the significant coefficients of interaction terms (AB, AC, and BC). Although there may be some correlation between the factors, the VIF values (>1) stay within reasonable bounds, suggesting no significant multicollinearity.

### 2.6. Selection of the Optimum NIT-TECs

When there are several responses to take into account at once, the desirability function is a crucial optimization tool for determining the optimal combination of formulations or process variables. The optimum TEC was selected based on desirability as previously stated, with a value of 0.828 (Figure 2D). The optimum TEC was fabricated utilizing 30 mg fenchone, 30 mg ceramide, and 0.5% CS, having an EE% of 98.87 ± 0.69%, PS of 129.43 ± 5.43 nm, PDI of 0.433 ± 0.022, and ZP of −25.99 ± 0.99 mV. The optimized formulation was validated experimentally, and the observed results were in good agreement with the predicted values, confirming the reliability and robustness of the optimization process. The agreement between expected and experimental data validates the suitability of the underlying model from a statistical validation perspective. The absence of a significant discrepancy (*p* > 0.05) between the observed and predicted responses indicates the robustness and validity of the model.

The composite desirability rating of 0.828 that the numerical optimization produced is typically regarded as a high degree of global optimization efficiency (values > 0.8 are seen to indicate an excellent compromise among responses). According to statistics, this number indicates that the chosen formulation minimally deviates from ideal aims while satisfying all given limitations. Maximizing EE%, minimizing PS and PDI (for uniform nanoscale dispersion), and keeping a suitably high negative ZP (for colloidal stability) were probably the optimization criteria.

### 2.7. In Vitro Drug Release

**Figure 3:**
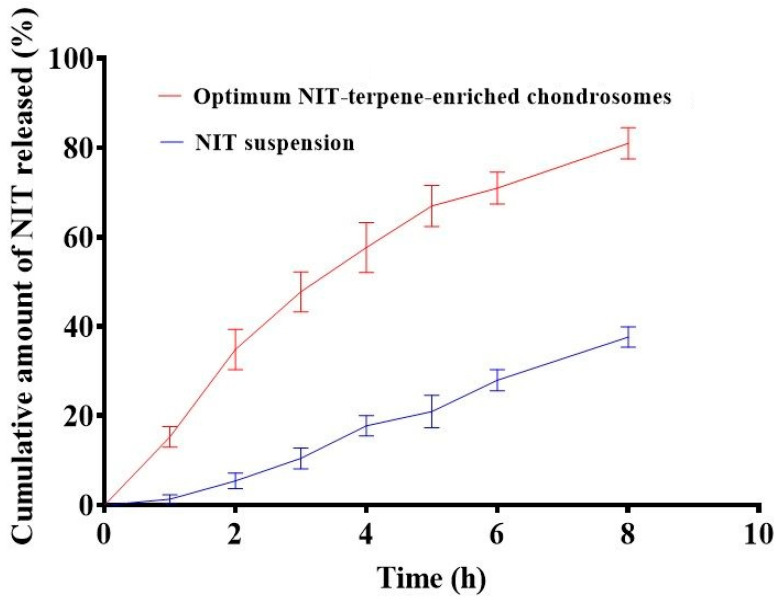
In vitro release study for the NIT suspension and the optimum formulation (*n* = 3).

Nanocarriers are engineered particles, typically 10–1000 nm in size, that serve as advanced delivery systems. They encapsulate poorly soluble drugs, improving their dispersion and modifying their interaction with the lung environment to enhance therapeutic performance. NTZ is an extremely poorly soluble drug with a solubility of 0.00755 mg/mL [35]. Figure 3 shows that the amount of drug released from the NIT suspension and the optimum TEC were 37.67 ± 2.30 and 80.99 ± 3.50, respectively. It is obvious from Figure 3 that the release of NIT from the TECs is significantly (*p* ≤ 0.05) faster than that from the NIT. This could be ascribed to the solubilization of NIT by the PC carrier [36]. It is well known that the PC has surface-active aspects, which has led to improved solubility of the encapsulated poorly soluble drug, NIT [37]. Furthermore, the hydrophilic character of CS is expected to facilitate NIT release from the TEC system by raising its concentration [38]. The faster release of NIT may be due to increased hydration and opening of the hydrophilic matrix with increased penetration of water, leading to dissolution of the drug [39]. According to the calculated values of the regression coefficient, the Higuchi model, which suggests that drug release is controlled and proceeds via diffusion from the developed system, best expressed the in vitro release profile of NIT from the optimum TEC formulation (r^2^ = 0.972). The kinetic model parameters are presented in Table 3.

### 2.8. Transmission Electron Microscopy (TEM)

**Figure 4:**
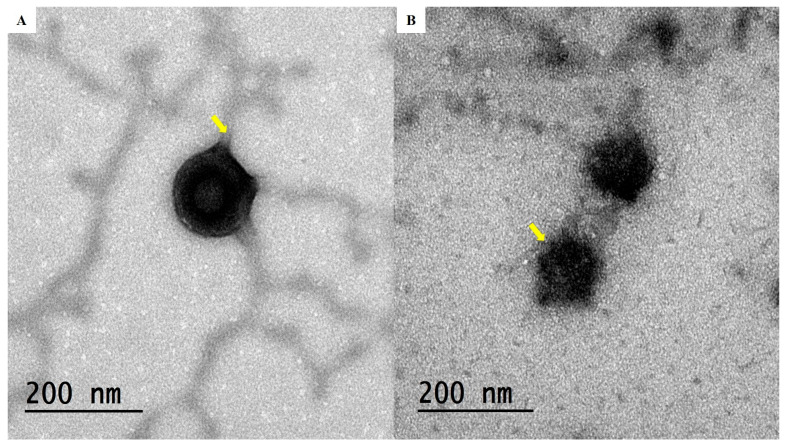
Transmission electron microscope micrograph for the optimum TEC (**A**,**B**). Yellow arrow represent bulbous feature.

When ceramide was added to TECs, the vesicular curvature changed from spherical to tubular, as depicted in the TEM pictures (Figure 4A,B) of the optimum TEC, which display the typical vesicular form of cerosomes. Ceramide incorporation increased bilayer rigidity and altered curvature, producing the characteristic tubular cerosome morphology. The greater packing parameter of ceramide (1.2) compared to PC (0.7) further enhanced this effect by producing a more flattened PC bilayer curvature. Additionally, the tubules’ distinctive bulbous feature was displayed, where a small number of spherical nanovesicles were occasionally seen beside the tubules. This distinguishing property was previously described [33] because ceramides have a propensity to disperse across the vesicular bilayer membrane unevenly, leading to the development of round-shaped, ceramide-poor vesicles and flat-shaped, ceramide-rich vesicles.

### 2.9. Scanning Electron Microscopy (SEM)

**Figure 5:**
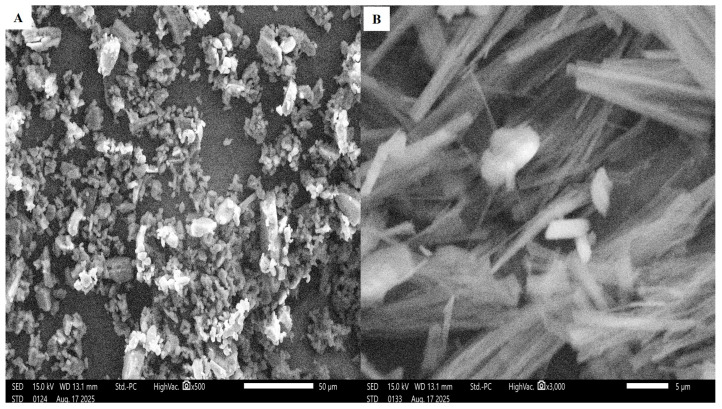
Scanning electron microscope images for the (**A**) pure NIT and (**B**) optimum TEC.

The SEM images of the lyophilized TEC compared to the drug powder (Figure 5A) exhibit the elongated rod-shaped fragments of the nanovesicles (Figure 5B). According to a prior study by Shukla et al. [40], changing the shape of the particle from spherical to elongated (rod-like) would improve its aerodynamic features and decrease phagocytic uptake and macrophage internalization, indicating that it could be successfully used through inhalation. The superior aerodynamic properties of the rod-like particles were attributed to their ability to align with the airflow direction. This alignment minimized deposition in the upper respiratory tract and instead promoted deposition in the peripheral airways.

### 2.10. Differential Scanning Calorimetry (DSC)

**Figure 6:**
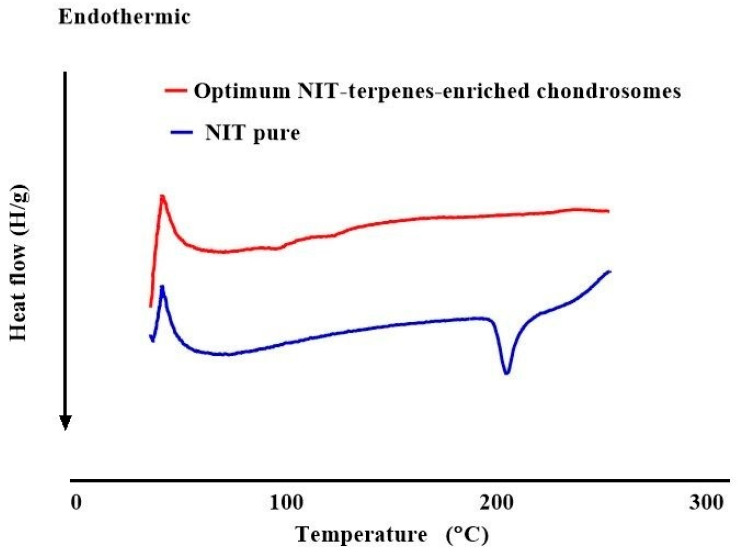
Differential scanning calorimetry study for pure NIT and the optimum formulation.

DSC is essential for understanding the thermal behavior, stability, compatibility, and physical state of components in nanoparticle systems. The pure NIT (Figure 6) showed a melting peak at 210 °C, proving its crystallinity [41]. The optimum TEC showed the disappearance of the melting point of NIT. Further, DSC analysis confirmed that NIT was transformed from a crystalline to an amorphous form in the optimum TEC [42].

### 2.11. FT-IR Spectroscopy

**Figure 7:**
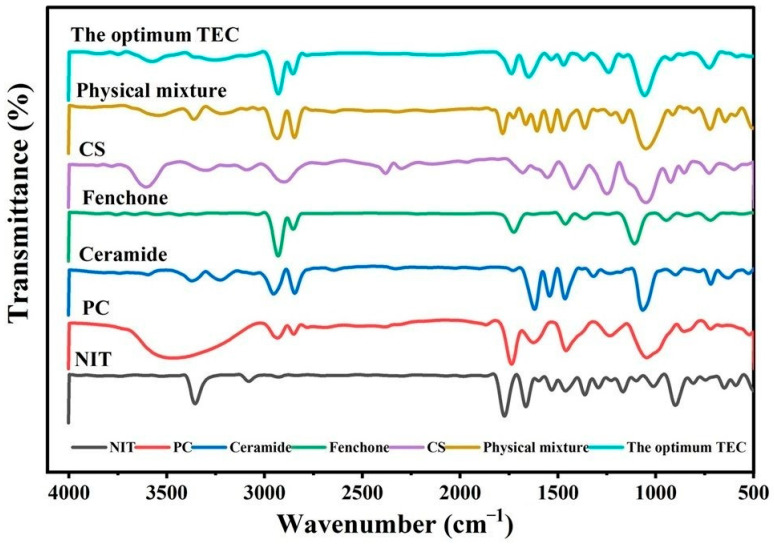
FT-IR spectra of NIT, the excipient, the physical mixture, and the optimum formulation.

The FT-IR spectrum of NIT (Figure 7) exhibited characteristic absorption bands at 3350.20 cm^−1^ corresponding to aromatic N–H stretching, 1520.34 cm^−1^ attributed to C–N-H bond stretching, 1771.04 cm^−1^ corresponding to the carbonyl group (C=O), 1465.35 cm^−1^ belonging to the C=C aromatic group, and 1355.35 cm^−1^ due to N=O stretching attached to the aromatic ring [43]. Furthermore, comparison of the FT-IR spectra of NIT with those of its physical mixture revealed the presence of all the characteristic absorption bands of the drug within the mixture, suggesting the absence of any significant chemical interactions between NIT and the formulation excipients. In contrast, the characteristic drug bands disappeared from the IR spectrum of the optimum TECs, suggesting efficient encapsulation of the drug within the nanovesicles [44].

### 2.12. Effect of Short-Term Storage

The assessment of the stocked TECs revealed an EE% of 97.00 ± 1.00%, PS of 131.00 ± 1.00 nm, PDI of 0.467 ± 0.001, and ZP of −26.40 ± 0.20 mV compared to the freshly prepared formulation, which showed no significant differences (*p* > 0.05). This good stability might be attributed to the existence of long saturated hydrocarbon chains of ceramide, which reduce membrane fluidity, preventing nanoparticle deformation, fusion, or leakage of the payload [45].

### 2.13. pH Measurement

Pulmonary application requires isotonic formulations with a pH value between 3 and 8.5 [46]. The pH of the formulated optimum TEC ranged from 6.50 ± 0.01 to 7.31 ± 0.001, suggesting the applicability of utilizing the formulation in the lung.

### 2.14. Mucin Test

For the treatment of infectious acute lung illnesses, the development of nanoformulations with easy preparation and mucoadhesive qualities for medication transport to the lungs is crucial [47]. The ZP of the pure mucin solution was −4.70 ± 0.10 mV. At the same time, the ZPs of the optimum formula before and after mixing with the mucin were −25.99 ± 0.99 and −19.15 ± 0.02 mV, respectively. The ZP of the mucin solution shifted to a more negative value due to the contribution of surface negative charges of CS and ceramide. This result confirmed the possibility of the optimum TECs adhering to the lung [48].

### 2.15. Aerodynamic Particle Size Characterization

The amount of NIT was determined by a validated high-performance liquid chromatography (HPLC) method. The method demonstrated excellent linearity over the concentration range of 2–100 µg mL^−1^ (r^2^ = 0.9999). The limit of detection (LOD) and limit of quantification (LOQ) were reported as 0.1 µg mL^−1^ and 0.4 µg mL^−1^, respectively, at an injection volume of 20 µL. The specificity and stability-indicating capability of the method were confirmed under stress conditions. Precision was also acceptable, with relative standard deviation (RSD) values of less than 1.0% for intra-day and 0.7% for inter-day measurements, while the accuracy showed an RSD of 0.3% [49].

**Figure 8:**
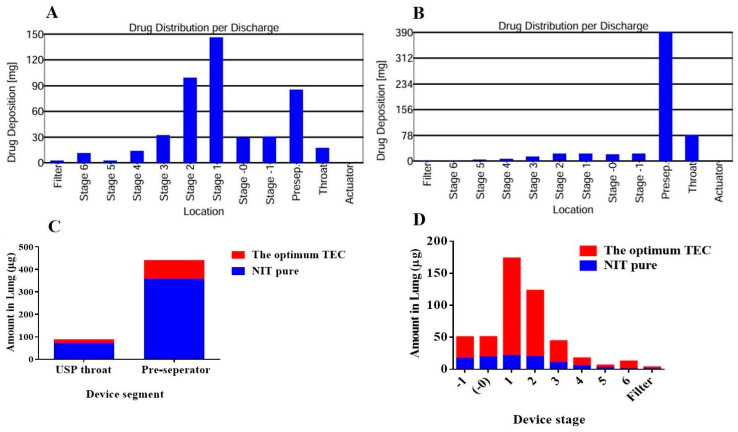
Drug deposition of (**A**) pure NIT, (**B**) the optimum formulation in the different stages of the cascade impactor, (**C**) the device segment, and (**D**) the device stage.

In comparison to NIT powder, the optimal TEC produced a significantly (*p* ≤ 0.5) higher total emitted dose (TED), fine particle dose (FPD), and fine particle fraction (FPF) and a lower mass median aerodynamic diameter (MMAD), indicating much greater flow and consequently better aerosolization and lung deposition. The reduced MMAD values of the optimum TEC (3.08 ± 0.02 µm) may contribute to improved deep deposition in the lung, particularly within the bronchial airways. The optimal TEC’s lower MMAD compared to pure NIT MMAD (5.18 ± 0.02 µm) may ensure deposition in the alveolar region [50]. Additionally, it was previously established that in order to achieve central and deep lung deposition, the ideal MMAD for pulmonary delivery should be between 1 and 3 µm [51]. Consequently, the optimum TEC aerosol formulation of NIT may enhance pulmonary deposition relative to pure NIT, aligning with previous research [52]. In conclusion, the optimum TEC aerosol formulation shows promise for improved pulmonary delivery over the pure drug, a premise that should be validated through a clinical bioequivalence study to consolidate this finding [53]. Compared to the pure NIT, the optimum TEC formulation demonstrated statistically superior performance, with significantly (*p* < 0.05) greater TED and FPD. The TED values for the optimum TEC compared to pure NIT were 520.26 ± 40.11 µg and 430.53 ± 8.02 µg, respectively, whereas the calculated FPD values were 215.71 ± 12.55 and 43.21 ± 2.81, respectively. The results obtained indicate the importance of improving the aspects of NIT using the optimum TEC. The emitted dose from NIT powder was greater than that from the optimum TECs, which is attributed to the utilized volume of NIT-loaded TECs comprising NIT powder and many other excipients. Figure 8 reveals the superiority of the improved NIT in being enclosed within the USP throat and pre-separator and deposited deeper in the tissues of the lung. The total amount of the optimum TEC enclosed within both the pre-separator and the USP throat was lower compared to the free NIT (*p* < 0.05). Free NIT indeed reached stage 4 in the Andersen cascade impactor; however, the amount of the optimum TECs was significantly greater (*p* < 0.05). Only the optimum TEC managed to deposit much deeper in the Andersen cascade impactor.

### 2.16. In Vitro Cytotoxicity

**Figure 9:**
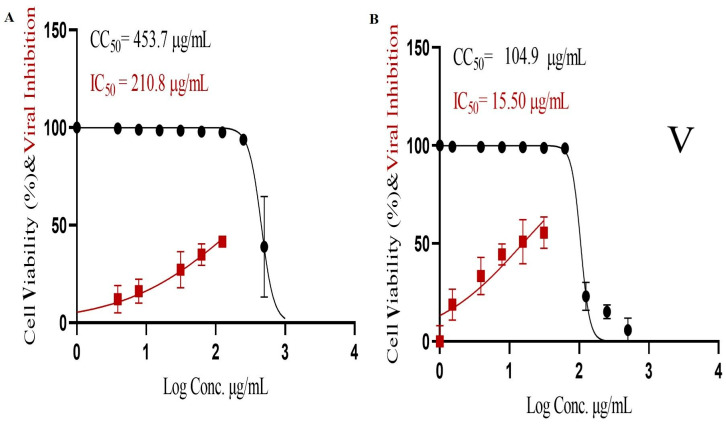
The effects of NIT (**A**) and the optimum formulation (**B**) on the viability of SARS-CoV-2.

The results showed that optimum TEC had high antiviral activity against SARS-CoV-2, as presented in Figure 9. The concentration had 50% cytotoxicity (CC_50_) = 104.90 µg/mL, and inhibitory concentration 50 (IC_50_) = 15.50 µg/mL, while the pure NIT had CC_50_ = 453.70 µg/mL and IC_50_ = 210.80 µg/mL. The improved activity of NIT-loaded optimum TEC was attributed to the composition of the formulation: fenchone, CS, and ceramides. Fenchone shows greater antimicrobial activity because of its richness with phenolic and alcoholic compounds and has proven activity against COVID-19 [10]. Further, ceramides hinder the viruses’ ability to attach to the host cells as well as their replication [16]. Further, previous investigation stated that CS, with its anti-inflammatory effect together with its inhibition of SARS-CoV-2 Mpro activity, should be quite beneficial for the prevention and treatment of COVID-19 due to its ability to impair viral binding, reduce inflammation, and protect against vascular complications [54].

### 2.17. In Silico Study

**Figure 10:**
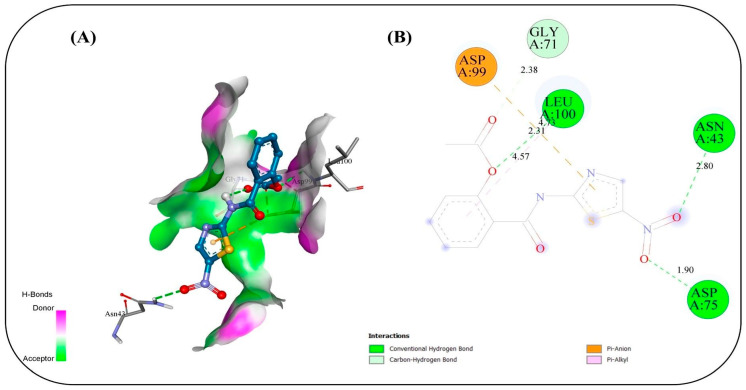
Binding interactions between the copper triflupromazine serine and the active site of methyl transferase: (**A**) 3D interaction visualization, (**B**) 2D interaction map.

**Figure 11:**
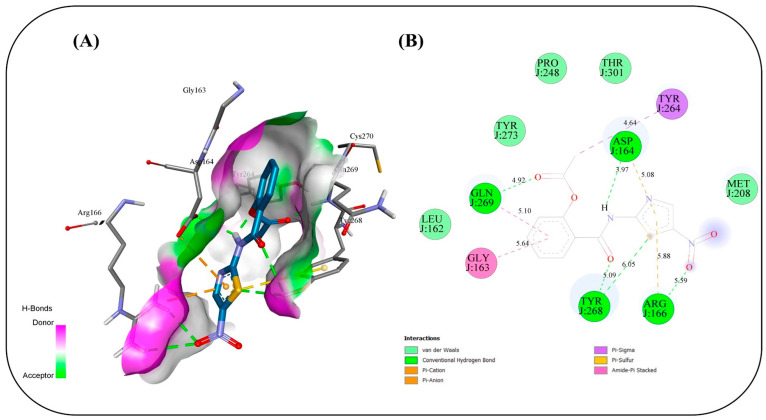
Binding interactions between the copper triflupromazine serine and the active site of PLpro: (**A**) 3D interaction visualization, (**B**) 2D interaction map.

**Figure 12:**
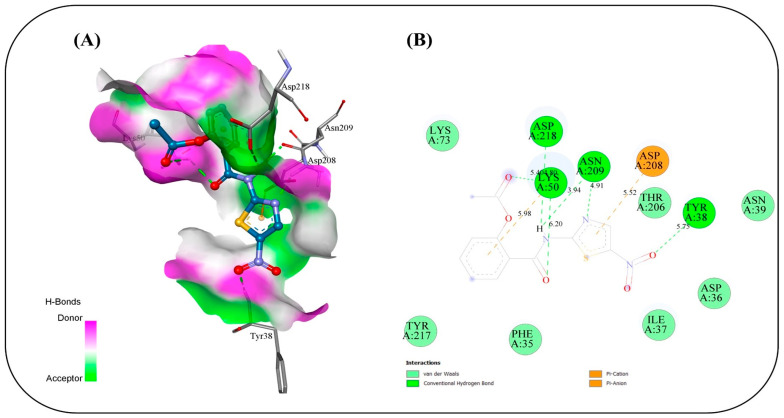
Binding interactions between the copper triflupromazine serine and the active site of RdRp: (**A**) 3D interaction visualization, (**B**) 2D interaction map.

**Figure 13:**
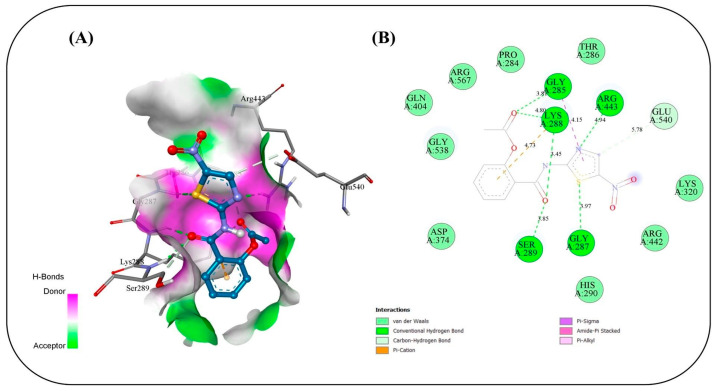
Binding interactions between the copper triflupromazine serine and the active site of helicase: (**A**) 3D interaction visualization, (**B**) 2D interaction map.

**Figure 14:**
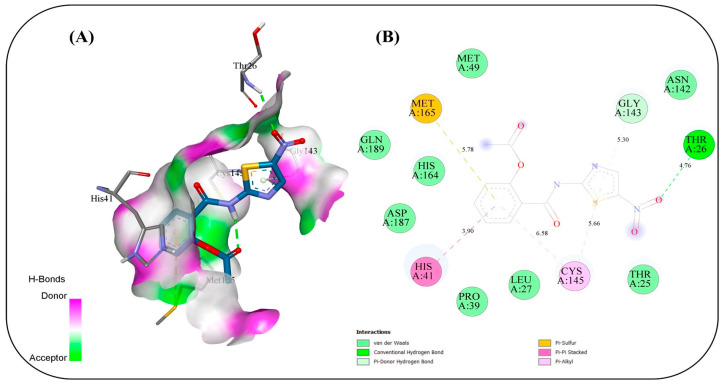
Binding interactions between the copper triflupromazine serine and the active site of main protease (Mpro): (**A**) 3D interaction visualization, (**B**) 2D interaction map.

Molecular docking simulations were conducted to evaluate the binding interactions of NIT and its four co-formulated components CS, ceramide, fenchone, and PC against five key SARS-CoV-2 target proteins: methyl transferase (PDB ID: 6YZ1), (PLpro, PDB ID: 7JRN), (RdRp, PDB ID: 7ED5), helicase (PDB ID: 5RMM), and main protease (Mpro, PDB ID: 7L8J) (Table 4). The docking results provide only a preliminary indication of potential molecular interactions and binding tendencies based on their predictive nature. Docking of NIT with the methyl transferase revealed a strong binding affinity of −7.2 kcal/mol, stabilized by three key conventional hydrogen bonds with ASN A:43 (2.80 Å), ASP A:75 (1.9 Å), and LEU A:100 (2.314 Å), along with supporting carbon–hydrogen, Pi–anion, and Pi–alkyl interactions. Among the co-formulated compounds, CS displayed the highest binding affinity (−7.8 kcal/mol), followed by ceramide, fenchone, and PC, suggesting potential synergistic engagement with this target (Figure 10). For PLpro, NIT suggested a binding affinity of −7.2 kcal/mol, forming a dense interaction network involving seven hydrogen bonds with residues ASP164, ARG166, TYR268, and GLN269, alongside Pi–cation, Pi–anion, and amide–Pi stacked interactions that further stabilized the complex. The co-formulated compounds also exhibited favorable binding energies, with fenchone (−6.3 kcal/mol) and CS (−6.2 kcal/mol) showing the most notable interactions (Figure 11). Docking to the RNA-dependent RNA polymerase (RdRp) indicated the highest affinity among all targets, with NIT achieving −8.0 kcal/mol. The binding pose was stabilized by a network of seven hydrogen bonds involving ASN209, ASP218, TYR38, and LYS50, in addition to electrostatic Pi–cation and Pi–anion interactions. The co-components also showed promising affinities, led by CS (−7.6 kcal/mol), implying their collective contribution to target inhibition (Figure 12). Interaction analysis with the helicase protein yielded a binding affinity of −7.6 kcal/mol, supported by six conventional hydrogen bonds with residues such as GLY A:285, GLY A:287, LYS A:288, ARG A:443, and SER A: 289 along with carbon–hydrogen bonds and Pi–cation, Pi–sigma, and amide–Pi stacked interactions. The other compounds showed notable affinities, especially CS (−7.8 kcal/mol), reinforcing its potential synergistic effect (Figure 13). Finally, docking against the main protease (Mpro) revealed a binding affinity of −6.6 kcal/mol for NIT, with key hydrogen bonds involving THR A:26 as well as hydrophobic Pi stacked and Pi–alkyl interactions with HIS A:41 and CYS A:145, and a distinctive Pi–sulfur interaction with MET A:165. Among the additional compounds, CS once again exhibited the strongest binding (−7.9 kcal/mol), followed by ceramide, PC, and fenchone (Figure 14). Collectively, these results highlight that NIT and its co-formulated components display significant binding affinities across all five SARS-CoV-2 targets, particularly toward RdRp, helicase, and methyl transferase, supporting the hypothesis of a multi-target and potentially synergistic antiviral mechanism. The observed binding affinities support a possible multi-target interaction profile, which may contribute to antiviral effects, but requires further validation through in vivo studies.

The molecular docking simulations revealed that NIT and its co-formulated components CS, ceramide, fenchone, and PC exhibit substantial interactions with multiple SARS-CoV-2 target proteins, underscoring their potential as multi-target antiviral agents. The consistent binding across all five studied viral proteins (methyl transferase, PLpro, RdRp, helicase, and Mpro) supports the possibility that this formulation may interact with multiple viral targets, which could be relevant for antiviral activity, pending experimental validation on essential viral replication and transcription processes. Among all targets, the RNA-dependent RNA polymerase (RdRp) displayed the highest affinity for NIT (−8.0 kcal/mol), suggesting potential interaction with the active site that may be relevant to viral RNA synthesis. The dense hydrogen-bonding network involving residues ASN209, ASP218, TYR38, and LYS50, complemented by electrostatic interactions, suggests a stable and specific binding mode that could impede the enzyme’s catalytic activity. The high affinity of CS (−7.6 kcal/mol) toward the same site reinforces the likelihood of a synergistic inhibitory effect when combined. Similarly, the methyl transferase complex showed a strong interaction with NIT (−7.2 kcal/mol), primarily stabilized by hydrogen bonds and hydrophobic contacts. Given the enzyme’s role in RNA capping and evasion of host immune recognition, interaction at this site may influence viral mRNA processing, although this cannot be confirmed from docking alone. The even higher affinity of CS (−7.8 kcal/mol) implies it may further enhance this effect. For the helicase target, NIT suggested a robust binding affinity (−7.6 kcal/mol), forming multiple hydrogen bonds with key active-site residues. This interaction occurs near residues involved in ATP binding, suggesting possible effects on helicase function essential for viral genome replication. Again, CS exhibited comparable affinity (−7.8 kcal/mol), suggesting a reinforcing interaction pattern. In contrast, docking against the PLpro and Mpro revealed relatively moderate affinities (−7.2 and −6.6 kcal/mol, respectively), though the presence of multiple hydrogen bonds and π-type interactions indicates that NIT still maintains significant binding stability. These proteases are critical for processing viral polyproteins; thus, even moderate inhibition may contribute to an overall antiviral effect when combined with potential interference at other targets. Across all proteins, CS consistently showed the highest binding affinities among the co-formulated compounds, suggesting that it may play a supportive or synergistic role in enhancing the overall antiviral efficacy of NIT. The recurring presence of stabilizing hydrogen bonds, π–π, and hydrophobic interactions across different binding pockets highlights the versatility of the compound mixture in engaging multiple viral sites. Taken together, these findings might suggest that NIT and its co-formulated components might have their antiviral potential due to a multi-target inhibitory mechanism, simultaneously engaging several key enzymes essential for SARS-CoV-2 replication and transcription. This multifaceted mode of action may offer a multi-target interaction profile, which warrants further investigation, offering a pharmacologically advantageous strategy in the management of COVID-19.

## 3. Materials and Methods

### 3.1. Materials

NIT was provided by UTOPIA Pharm. Co. (Cairo, Egypt). Phospholipid (PC; purity ~95–99%) and mucin from porcine stomach type II were supplied by Sigma-Aldrich (St. Louis, MO, USA). CS was sourced from EPICO Pharm. Co. (Cairo, Egypt). Fenchone (purity 99%) and 1,8-cineole (purity 98%) were obtained from Alfa Aesar (GmbH, Karlsruhe, Germany). Ceramide III was supplied by Evonik Industries (GmbH, Essen, Germany). Mannitol and glycine were purchased from El-Nasr Pharm. Co. (Cairo, Egypt). HPLC-grade acetonitrile and ethanol were obtained from Merck (Darmstadt, Germany).

### 3.2. TECs Loaded with NIT Preparation

TECs loaded with NIT (10 mg) were fabricated via ethanol injection. Firstly, PC (100 mg), NIT, terpenes (10 mg of cineole or fenchone), CS, and ceramide III were liquefied in 2 mL of ethanol [55]. The organic mixture was injected slowly into heated (60 °C) distilled water (10 mL), stirred at 1500 rpm for 30 min on a stirrer (MSH-20D, GmbH, Wertheim, Germany), and then kept at 4 °C overnight.

### 3.3. EE% and DL%

The vesicular dispersion was centrifuged for 1 h at 20,000 rpm and 4 °C using a cooling centrifuge (Sigma 3K 30, Osterode am Harz, Germany). The NIT concentration was determined at λ_max_ 345 nm [56] using a UV-VIS spectrophotometer (Shimadzu UV1650, Kyoto, Japan). A calibration curve was constructed by preparing standard NIT solutions over a defined concentration range, and the absorbance was measured at 345 nm.

EE% was determined using the following equation [45]:

(1) $$\mathrm{EE}\%\;=\;\left(\frac{Total\;NIT\;amount-Unentrapped\;NIT}{Total\;NIT\;amount}\right)\;\times \;100$$

The drug loading capacity (DL%) was calculated using the following equation [57]:

(2) $$\mathrm{DL}\%\;=\;\left(\frac{Entrapped\;NIT\;amount}{Total\;TECS\;amount}\right)\;\times \;100$$

### 3.4. PS, PDI, and ZP

A zetasizer (Malvern, UK) was employed to assess the PS and PDI of the TECs utilizing a light-scattering technique. Also, it was employed to determine the ZP of TECs by detecting their electrophoretic motion [58].

### 3.5. D-Optimal Design Construction

The impact of the factors in producing TECs was studied utilizing a D-optimal design through operating Design expert^®^ software (Stat Ease, Minneapolis, MN, USA). Factors were terpene type (X_1_), ceramide III amount (X_2_), and CS concentration (*v*/*v*) (X_3_). Responses were EE% (Y_1_), PS (Y_2_), and ZP (Y_3_) (Table 5). A solution with the smallest PS and the highest EE% and ZP was attained in the optimization stage.

### 3.6. In Vitro Release

The USP dissolution apparatus II (Pharma Test, Hainburg, Germany) was used to evaluate in vitro release at 37 °C for 6 h. An amount of 1 mL of the optimum TECs and NIT suspension equivalent to 1 mg was put into tubes with a 0.706 cm^2^ area. Phosphate buffer (50 mL) (pH 7.4) with 1% Tween 80 was used as the receptor medium. The drug solubility in the receiver medium was 0.5 mg/mL and the volume used (50 mL) ensured maintenance of sink conditions. Samples were taken at 1, 2, 3, 4, 6, and 8 h [59]. Release studies were conducted using three independent samples for each formulation. Further, the data from the release experiment were fitted to various kinetic models, including zero, first-order, Higuchi, Korsmeyer–Peppas, and Hixson–Crowell models [60], to determine the drug release mechanism of NIT from the TECs according to the best-fit model with the highest r^2^.

### 3.7. TEM

The optimum TEC morphology was identified by TEM (Joel JEM, Tokyo, Japan) [61]. A sample that had been stained with phosphotungstic acid (2% *v*/*v*) was placed on a copper–carbon grid [62].

### 3.8. DSC Study

The optimum TEC was frozen and then lyophilized, and the pure NIT and lyophilized TEC thermograms were recorded, using a previously calibrated differential scanning calorimeter (Shimadzu DSC 50; Kyoto, Japan) after heating 3–4 mg samples of each in hermetically sealed flat-bottomed aluminum pans over a temperature range of 30 to 250 °C, at a constant heating rate of 10 °C /min, under an inert nitrogen flow of 30 mL/min [63].

### 3.9. FT-IR Spectroscopy

FT-IR analysis was performed for the individual components, including NIT, PC, ceramide, fenchone, CS, their physical mixture, and the optimum TEC using an FT-IR spectrophotometer (Bruker, Coventry, UK) [64]. This analysis aimed to detect any possible interactions between NIT and the formulation excipients. Spectra were recorded at 25 °C over 4000–500 cm^−1^ after carefully drying the samples and compressing them into potassium bromide (KBr) discs [65].

### 3.10. Effect of Storage

The effect of storage of the optimum TECs was investigated to monitor the physical change. The optimum TECs were stocked for 3 months at room temperature, and then their EE%, PS, PDI, and ZP results were compared with the results of a fresh one [66].

### 3.11. pH Measurement

To test the optimum TECs’ pulmonary tolerance, the pH was assessed by applying a potentiometer (Inolab, Weilheim, Germany) at room temperature [45].

### 3.12. Mucin Test

The mucoadhesive properties of the optimum TECs were examined using mucin (1% *w*/*v*) by mixing the optimum TEC with mucin. Before testing, the ZP values of both the mucin solution and the optimum TEC were assessed and subsequently compared to the ZP of the mixture formed [67].

### 3.13. Lyophilization of the Optimized TECs

The optimum TECs were kept for freezing at −20 °C for 24 h in an ultra-cold freezer (Revco™, Thermo Scientific, Waltham, MA, USA). The pre-frozen formula was then freeze-dried using a Flexi-Dry™ MP Freeze Dryer (SP Scientific, Gardiner, NY, USA) at −90 °C and pressure of 380 mT for 48 h to yield dry TECs [68]. Mannitol (10% *w*/*v*) and glycine (1% *w*/*v*), as cryoprotectants, were appended to the dispersions before freezing [69].

### 3.14. SEM

Samples from NIT and the lyophilized optimum TECs were attached to sample stubs using double-sided tape. Samples were covered with gold using sputter coating and then photographed under a voltage of 15 kV SEM at magnifications of 2500× to 5000× by SEM (JSM, JEOL, Tokyo, Japan) [70].

### 3.15. Simulated In Vitro Inhalation

#### 3.15.1. Aerodynamic Characterization

The optimum lyophilized TECs and NIT were placed in capsules (size 3), appropriate for the aerolizer (Novartis Pharma, Cairo, Egypt), to assess their aerodynamic characteristics.

#### 3.15.2. Aerodynamic Characterization

The Andersen MKII cascade impactor (ACI) was then assembled with modified collection plates for a flow of 60 L/min, where stages 0 and 7 were replaced by −0 and −1 on the top of the impactor [71]. The various plates were then coated with silicone fluid and allowed to dry for an hour before examination. The pre-separator was then filled with mobile phase (10 mL) and a GF 50 filter (Copley Scientific Ltd., Nottingham, UK). The aerolizer operated at a flow rate of 60 L/min, with a 4 s flow period to pull 4 L of air through the device’s mouthpiece. The flow was monitored by applying a digital flow meter (MKS Instruments, Andover, MA, USA). From each formula, two outcomes were obtained. A group of 7 NIT doses was chosen by random scheduling for each aerodynamic size determination. Three capsules per dose were individually inserted into the aerolizer and released into the ACI device. To administer a single dose, the inhaler was filled with a single capsule. The loaded aerolizer was positioned along the horizontal axis and firmly inserted into the ACI USP throat. The pump was switched on, allowing 4 L of air to be extracted from the inhaler during each determination. Following each step, the ACI was rinsed with a specified volume of the mobile phase mixture, which was made up of acetonitrile (45:55, *v*/*v*) and 0.1% o-phosphoric acid solution, pH 6.0 [49], whereas the filter from the final stage was fully immersed in the mobile phase mixture and sonicated for 3 min to remove any retained drug. The quantity of drug collected on each stage and the final filter was quantified utilizing the HPLC technique. TED represents the fraction of the loaded dose that successfully reached the ACI; fine particle dose (FPD; particles less than 5 µm), FPF, and MMAD were detected by applying Copley Inhaler Testing Data Analysis Software, Version v2.4.0 (CITDAS, Copley Scientific, Nottingham, UK).

### 3.16. In Vitro Cytotoxicity

#### 3.16.1. MTT Cytotoxicity Assay

The cytotoxicity of optimum TECs was examined in vitro in Vero-E6 cells using the 3-(4,5-dimethylthiazol-2-yl)-2,5-diphenyltetrazolium bromide (MTT) technique. The cells were seeded and incubated for 24 h at 37 °C in 5% CO_2_. After 24 h, cells were handled in triplicate with different doses of the tested dispersion. After 24 h, the supernatant was removed, and the monolayers were rinsed with buffer. MTT solution (20 µL of 5 mg/mL stock solution) was applied to each well three times and incubated at 37 °C for 4 h, followed by medium aspiration. An amount of 200 µL of acidified isopropanol was added to each well to dissolve the generated formazan crystals. The absorbance of the formazan solutions was measured at λ_max_ 540 nm using 620 nm as a reference wavelength using a multi-well plate reader. The percentage of cytotoxicity compared to the untreated control is as follows [72]:

(3) $$\%\;cytotoxicity=\frac{absorbance\;of\;control\;cells-absorbance\;of\;treated\;cells}{absorbance\;of\;control\;cells}\;\times \;100$$

The plot of % cytotoxicity versus sample concentration was used to determine the concentration with 50% cytotoxicity (CC_50_).

#### 3.16.2. Inhibitory Concentration 50 (IC_50_) Determination

In 96-well tissue culture plates, 2.4 × 10^4^ Vero-E6 cells were distributed and cultured overnight in a humidified 37 °C incubator with 5% CO_2_. The cell monolayers were then washed once with buffer before being exposed to viral adsorption (hCoV-19/Egypt/NRC-03/2020 (GSAID accession number: EPI_ISL_430820)) for 1 h. The cell monolayers were then coated with 100 μL of DMEM bearing varied formulation concentrations. Subsequent to incubation at 37 °C in 5% CO_2_ incubator for 72 h, the cells were fixed and stained with 0.1% crystal violet in distilled water for 15 min. The optical density of the color was evaluated by applying an Anthos Zenyth 200RT plate reader (Anthos Labtec- Heerhugowaard, Heerhugowaard, the Netherlands) at 570 nm. The compound’s IC_50_ is the concentration needed to lower the viral cytopathic effect by 50% compared to the virus control [73].

### 3.17. In Silico Study

#### Computational Methods

The 3D structures of methyl transferase, papin-like protease (Plpro), RNA-dependent RNA polymerase (RdRp), helicase, and main protease (Mpro) proteins were retrieved from the RCSB Protein Data Bank via their corresponding PDB IDs (6WZ1, 7JRN, 7ED5, 5RMM, and 7L8J, respectively). The binding pocket was identified using the CASTpFold server [74], which predicts ligand-binding sites through structure-based geometric analysis methods. The protein structure was prepared by using AutoDockTools (Version 1.5.7) [75]. Missing residues were modeled with Modeller 10.6. The ligands were optimized into a 3D structure via Open Babel [76], and energy-minimized using the UFF and MMFF94 force field in RDKit [77]. Molecular docking simulations were performed in AutoDock vina with a grid box centered on the CASTpFold-predicted binding sites of the methyl transferase, papin-protease (Plpro), RNA-dependent RNA polymerase (RdRp), helicase, and main protease (Mpro) proteins, respectively: (coordinates: x = 86.734, y = 16.03, z = 32.587; dimensions: 56 × 42 × 26 Å), (coordinates: x = 13.852, y = 66.726, z = −0.714; dimensions: 40 × 40 × 40 Å), (coordinates: x = 117.198, y = 145.242, z = 85.849; dimensions: 44 × 44 × 44 Å), (coordinates: x = −17.46, y = 20.333, z = −52.749; dimensions: 100 ×100 × 100 Å), and (coordinates: x = 16.258, y = 15.604, z = 26.894; dimensions: 40 × 40 × 40 Å). The exhaustiveness value was set to 32 to ensure an extensive exploration of the binding site, while the number of output poses (num_modes) was set to 10 to allow sufficient conformational diversity. The energy range was maintained at 8.0 kcal/mol to include a wide variety of binding modes within a reasonable energy window. Docking calculations were performed utilizing 8 CPU cores to accelerate the computational process without compromising the quality of the results. Ligand–receptor interactions were analyzed and visualized using Discovery Studio 2025.

### 3.18. Statistical Analysis

Statistical analyses were performed using SPSS^®^ software version 22.0 (SPSS Inc., Chicago, IL, USA). For comparisons between two groups, either a paired *t*-test for the effect of storage or unpaired Student’s t-test was applied for in vitro release and aerodynamics, as appropriate. For comparisons among more than two groups (in the experimental design), one-way ANOVA was performed, followed by Tukey’s post hoc multiple comparison test. Further, the error bars in the manuscript represent the standard deviation.

## 4. Conclusions

In the present work, NIT (a repurposed antiviral drug) was fabricated in TECs utilizing the ethanol injection method. A D-optimal design was used to determine the impact of formulation variables on the vesicular aspects and to determine the optimum NIT-TEC. TECs were then formulated utilizing different quantities of ceramide and chondroitin sulfate, with various terpene types in the TEC constructs. The optimum TEC was the formulation prepared using 30 mg fenchone, 30 mg ceramide, and 0.5% CS, having an EE% of 98.87 ± 0.69%, PS of 129.43 ± 5.43nm, PDI of 0.433 ± 0.022, and ZP of −25.99 ± 0.99 mV. The optimum TEC revealed a significantly greater release pattern compared to the NIT. The aerodynamic results supported the notion that the optimal formula was an effective platform for pulmonary delivery and deep lung deposition of NIT. The in vitro cytotoxicity of NIT-loaded TEC revealed greater CC50 and lower IC50 values against normal Vero-E6 and virus-infected cells, respectively, compared to NIT. The antiviral activity of NIT and formulation ingredients against SARS-CoV-2 and its target enzymes was determined by applying computational studies. Therefore, the optimum TEC represents a nanocarrier for improving the antiviral potential of NIT.

## Figures and Tables

**Table 1:** Output data of the D-optimal analysis of nitazoxanide-loaded terpene-enriched chondrosomes.

Responses	EE (%)	PS (nm)	ZP (mV)
Adequate precision	24.74	32.73	76.87
Adjusted R^2^	0.910	0.984	0.996
Predicted R^2^	0.972	0.910	0.990
Significant factors	(X_1_ and X_3_)	(X_1_ and X_3_)	(X_1_, X_2_, and X_3_)

EE%: entrapment efficiency percentage; PS: particle size; and ZP: zeta potential.

**Table 2:** Experimental runs, independent variables, and measured responses of the D-optimal design of nitazoxanide-loaded terpene-enriched chondrosomes.

Formulation Code	Terpene Type	Ceramide Amount (mg)	Chondroitin Sulfate Concentration (*v*/*v*)	DL (%)	EE%	PS (nm)	PDI	ZP (mV)
F1	Cineole	10	0.25	62.18 ± 1.23	96.39 ± 0.67	200.07 ± 0.07	0.226 ± 0.009	−24.24 ± 0.79
F2	Cineole	10	0.25	61.27 ± 1.34	94.98 ± 0.65	178.99 ± 0.99	0.355 ± 0.10	−24.89 ± 0.13
F3	Cineole	30	0.25	58.69 ± 3.76	90.97 ± 0.09	150.44 ± 0.56	0.324 ± 0.023	−24.79 ± 0.19
F4	Cineole	30	0.25	59.69 ± 4.98	92.52 ± 1.45	147.99 ± 0.99	0.329 ± 0.028	−25.79 ± 1.19
F5	Cineole	10	0.5	57.73 ± 2.98	89.49 ± 0.51	314.95 ± 4.95	0.371 ± 0.003	−24.36 ± 0.49
F6	Cineole	30	0.5	57.57 ± 3.98	89.24 ± 0.74	340.49 ± 3.49	0.429 ± 0.015	−33.19 ± 0.37
F7	Fenchone	10	0.25	58.57 ± 4.98	90.79 ± 0.81	193.03 ± 4.83	0.385 ± 0.011	−28.49 ± 0.59
F8	Fenchone	30	0.25	59.99 ± 2.98	92.99 ± 0.01	183.04 ± 6.04	0.321 ± 0.02	−26.56 ± 0.69
F9	Fenchone	10	0.5	59.42 ± 2.23	92.11 ± 0.75	108.82 ± 0.82	0.391 ± 0.002	−20.09 ± 0.89
F10	Fenchone	10	0.5	59.82 ± 2.19	92.73 ± 1.15	115.99 ± 6.99	0.390 ± 0.001	−20.98 ± 1.78
F11	Fenchone	10	0.5	59.85 ± 1.87	92.78 ± 1.20	115.94 ± 7.94	0.380 ± 0.004	−21.59 ± 2.39
F12	Fenchone	30	0.5	63.78 ± 1.45	98.87 ± 0.69	129.43 ± 5.43	0.433 ± 0.022	−25.99 ± 0.99
F13	Fenchone	30	0.5	63.14 ± 3.87	97.88 ± 1.10	129.84 ± 5.14	0.375 ± 0.011	−27.39 ± 1.59
Optimum TEC observed results					98.87	129.43	0.433	25.99
Optimum TEC predicted results					98.81	129.00	0.432	25.00
Bias (%)					0.06	0.33	0.23	3.80

Data represented as mean ± SD (*n* = 3). EE%: entrapment efficiency percentage; PS: particle size; ZP: zeta potential; and PDI: polydispersity index.

**Table 3:** In vitro release kinetic model parameters of nitazoxanide-loaded terpene-enriched chondrosomes.

Model Name	Release Rate Constant (k)	Regression (R^2^)
Zero-order	10.22	0.930
First-order	−0.12	0.969
Higuchi	30.98	0.972
Korsmeyer–Peppas	0.79	0.960
Hixson–Crowell	0.25	0.965

**Table 4:** Summary of docking results.

Target Protein	Ligand	Binding Affinity (kcal/mol)	Key Interactions
Methyl transferase (6YZ1)	NIT	−7.2	H-bonds: ASN A:43, LEU A:100, and ASP A:75
			Pi–anion and Pi–alkyl interactions
	Sinefungin	−7.9	H-bonds: GLY A:73, LEU A:100, ASP A:114, TYR A:47, and ASN A:43
Papain-like protease (7jrn)	NIT	−7.2	H-bonds: ASP164, ARG J:166, TYR J:268, and GLN J:269
			Pi–cation, Pi–anion, and amide–Pi stacked interactions
	GRL0617	−9.9	H-bonds: GLN J:269, and ASP J:164
RNA-dependent RNA polymerase (7ed5)	NIT	−8	H-bond: ASN A:209, ASP A:218, TYR A:38, and LYS A:50
			Pi–cation and Pi–anion interactions
	AT-527	−8.9	H-bond: ASP A:208, LYS A:50, LYS A:73, ARG A:116, CYS A:53, ARG A:33, ARG A:55
Helicase (5RMM)	NIT	−7.6	H-bonds: GLY A:285, GLY A:287, LYS A:288, ARG A:443, and SER A: 289
			Carbon–hydrogen bonds, Pi–cation, Pi–sigma, and amide–Pi stacked interactions
	POB0066	−5.9	H-bonds: ASN B:516, SER B:486, and SER B:485
Main protease (7l8j)	NIT	−6.6	H-bonds: THR A:26
			Hydrophobic Pi stacked and Pi–alkyl interactions
	Rupintrivir	−8.4	H-bonds: GLU A:166, PHE A:140, SER A:144, and HIS A:163

**Table 5:** D-optimal design for optimization of nitazoxanide-loaded terpene-enriched chondrosomes.

Factors (Independent Variables)	Levels	
	Low (−1)	High (+1)
X_1_: Terpene type	Fenchone	Cineole
X_2_: Ceramide amount (mg)	10	30
X_3_: Chondroitin sulfate	0.25	0.5
Responses (Dependent Variables)	Constraints	
Y_1_: EE (%)	Maximize	
Y_2_: PS (nm)	Minimize	
Y_3_: ZP (mV)	Maximize (as absolute value)	

EE%: entrapment efficiency percent; PS: particle size; and ZP: zeta potential.

## Data Availability

The original contributions presented in this study are included in the article. Further inquiries can be directed to the corresponding author.

## Abbreviations

The following abbreviations are used in this manuscript:

ACI	Andersen MKII cascade impactor
CS	Chondroitin sulfate
DSC	Differential scanning calorimetry
DL%	Drug loading
EE%	Entrapment efficiency percent
FPD	Fine particle dose
FPF	Fine particle fraction
MMAD	Mass median aerodynamic diameter
MTT	3-(4, 5-dimethylthiazol-2-yl)-2, 5-diphenyltetrazolium bromide
NIT	Nitazoxanide
PS	Particle size
PDI	Polydispersity index
RSM	Response surface methodology
SEM	Scanning electron microscopy
TEC	Terpene-enriched chondrosomes
TED	Total emitted dose
TEM	Transmission electron microscopy
ZP	Zeta potential
